# Conservative Surgery of the Uterine Appendages

**Published:** 1911-05

**Authors:** Frank K. Boland

**Affiliations:** Atlanta, Ga.; Gynecologist, Wesley Memorial Hospital


### Journal-Record of Medicine

Successor to Atlanta Medical and Surgical Journal. Established 1855.
and Southern Medical Record. Established 1870.

OWNED BY THE ATLANTA MEDICAL JOURNAL CO.

Published Monthly

*Official Organ Fulton County Medical Society, State Examining Board, Presbyterian Hospital, Atlanta, Birmingham and Atlantic Railroad Surgeons' Association, Chattahoochee Valley Medical and Surgical Association, Etc.*

EDGAR G. BALLENGER., M. D., Editor.
BERNARD WOLFF, M. D., Supervising Editor.
A. W. STIRLING, M. D., C. M., D. P. H., J. S. HURT, B. Ph., M. D.
GEO. M. NILES, M. D., W. J. LOVE, M. D., (Ala.); Associate Editors.
E. W. ALLEN, Business Manager.

COLLABORATORS

Dr. W. F. WESTMORLAND, General Surgery.
F. W. McRAE, M. D., Abdominal Surgery.
H. F. HARRIS, M. D., Pathology and Bacteriology.
E. B. BLOCK, M. D., Diseases of the Nervous System.
MICHAEL HOKE, M. D., Orthopedic Surgery.
CYRUS W. STRICKLER, M. D., Legal Medicine and Medical Legislation.
E. C. DAVIS, A. B., M. D., Obstetrics.
E. G. JONES, A. B., M. D., Gynecology.
R. T. DORSEY, Jr., B. S. M. D., Medicine.
L. M. GAINES, A. B., M. D., Internal Medicine.
J. N. LeCONTE, M. D., Disease of the Stomach and Intestines.
L. B. CLARKE, M. D., Pediatrics.
EDGAR PAULIN, M. D., Opsonic Medicine.
THEODORE TOEPEL, M. D., Mechano Therapy.
R. R. DALY, M. D., Medical Society.
A. W. STIRLING, M. D., etc., Diseases of the Eye, Ear, Nose and Throat.
BERNARD WOLFF, M. D., Diseases of the Skin.
E. G. BALLENGER, M. D., Diseases of the Genito-Urinary Organs.

VOL. LVIII. MAY, 1911 NO. 2.

### CONSERVATIVE SURGERY OF THE UTERINE APPENDAGES.

FRANK K. BOLAND, M. D., ATLANTA, GA.

*Gynecologist, Wesley Memorial Hospital.*

It is the aim of every conscientious surgeon to preserve the sexual functions and instincts of his patients when he can do so without risk to life and health. We all appreciate the value of the uterine appendages in the animal economy, and yet I believe the after effects of their removal at times have been exaggerated. For instance, Giles, in a careful study of results in 162 consecutive cases of double salpingo-oophrectomy, collected the following statistics:

78% were in very good health afterwards, and a further 13%, though suffering in different ways, were better than before the operation, making in all 91% who were quite well, or who were improved. Indeed, he found that the general health after bilateral operation was better in a larger per cent. of cases than after unilateral operation. His experience also showed that it is better to leave the uterus, if possible, since trouble developed in it in only seven cases of 105 double salpingo-oophrectomies.

In 40% of the 162 cases menstruation continued, from which he judged that a portion of an ovary must have been left. The characteristics of artificial menopause observed by Giles after complete removal of ovaries and tubes were as follows:

Flushes of heat came on within three months of the operation in 80% of cases, and within a month in 55%. The average of duration of this phenomena was three or four years. 72% of the cases retained their bodily vigor and energy; 28% easily tired and complained of lack of energy. This result might be accounted for in part by the fact of an abdominal operation aside from the artificial menopause. 10% of the cases showed some degree of mental depression. One writer, quoting from Giles, states that he reported two cases of insanity, but I am unable to find this item in his figures.

In 68% of the cases the sexual instincts were increased or not affected; in 16% the sexual instincts were diminished, and in another 16% they were lost. Many of the patients showed a tendency to obesity, but not so much so as in the natural menopause, and in none of the cases were masculine characteristics developed, save where operation was done before or about the age of puberty.

These statistics should encourage us when we are called on to do a total extirpation of the adnexa, but, of course, it is true that another man's results might not be so comforting. As far as my experience has gone, it coincides very closely with the figures of Giles.

In discussing this subject, I wish to emphasize the prime importance of thorough preliminary treatment of inflammatory pelvic conditions. By this I mean the recumbent position, liquid diet, elimination, and the proper application of douches, stupes and other medical treatment. These things, carefully carried out, will greatly increase the number of cases amenable to conservative operations, and, indeed, will save some cases from the operating table altogether. Clark and Norris state that four to six weeks of normal temperature and blood counts should precede each operation. Accuracy of diagnosis will reduce the number of gynecological cases requiring emergency surgery to a minimum.

I do not believe there is very much to be done for an inflamed Fallopian tube except to take it out. A salpingitis of gonorreal origin, as most of them are, offers little chance for conservative surgery. The gonococcus is such a persistent organism that unless the part affected by it can be reached by direct treatment, the only way to cure it is by removal. But the other tube, if normal, must not be sacrificed. The liability of its becoming involved is very small, though I think this would depend largely on the condition of the endometrium. If both ovaries have been removed it is well to take both tubes also, but Kelly has reported a case of pregnancy occurring in a patient in whom the tube and ovary of opposite sides had been removed.

Except in the presence of malignant disease, a sound ovary should never be excised. Its retention preserves the internal secretion of this organ and softens the shock of an artificial menopause. Authorities differ as to the advisability of saving any sound part of an ovary. In case of one sound ovary and one greatly diseased ovary, it is safer to remove the diseased organ in toto, while in the case of one ovary hopelessly diseased, and the other containing some sound tissue, the question as to whether or not it is wise to attempt to preserve the healthy portion dpends upon several factors. In the first place, I believe the probability of cystic or other pathological condition developing in this portion hangs on the preservation of the blood supply in dealing with the affected part, and upon the position in which we leave the organ. Very often its nourishment and normal position are so interfered with in our effort to preserve its sound tissue that it becomes functionless, or gives the patient great discomfort, or falls easy prey to later disease. We are justified in taking this risk, however, in young women, and especially in those who desire motherhood, although some of the worst mental conditions have been produced by the removal of both ovarise in women past forty. The knowledge a woman has that it is not impossible for her to have a child may prevent a neureotic condition, whether she ever realizes her hope or not.

Giles' figures on 218 cases of salpingo-oophrectomy show that 33 patients, or 25% of the married women under forty became pregnant. Of these, 19 had full-time deliveries (some repeated), 5 had miscarriages, and seven had extra-uterine pregnancy, while two were pregnant when seen. From this, it appears, that the removal of the appendages of one side increases the liability to extra-uterine pregnancy. I have seen one such case, with Dr. J. L. Campbell.

In some cases of bilateral salpingo-oophrectomy the uterus should be left, and in others it is wiser to remove it. In an old case, with profuse irritating leucorrhea, the endometrium must be hopelessly diseased, and simple curretage will not cure it. In a more recent case, with less pronounced symptoms, preserving the uterus mitigates the chance of serious neurotic symptoms, and certainly it is a simpler and safer operation. Supravaginal hysterectomy is usually all that is indicated. The round ligaments should be stitched to the cervical stump.

Altogether, the subject of conservative surgery of the uterine appendages is one for which, like many others in medicine, it is hard to deduct fixed rules. There are so many exceptions that almost every case becomes a law to itself. Careful study of each individual case, accurate diagnosis, and painstaking, patient treatment will produce the best results.

